# Performance and emissions of diesel engine combustion lubricated with Jatropha bio-lubricant and MWCNT additive

**DOI:** 10.1038/s41598-025-86025-8

**Published:** 2025-01-20

**Authors:** Rajendra Uppar, P. Dinesha, Shiva Kumar

**Affiliations:** Department of Mechanical and Manufacturing Engineering, Manipal Institute of Technology, Manipal Academy of Higher Education, Manipal, 576104 India

**Keywords:** Biolubricant, Transesterification, Epoxidation, Jatropha, Friction power, Emission, Environmental sciences, Energy science and technology, Engineering, Nanoscience and technology

## Abstract

Vegetable oil-based lubricants, modified through transesterification and epoxidation, present a sustainable alternative to mineral lubricants for transport and industrial use. This study evaluates epoxidized jatropha oil (EJA) enhanced with multi-walled carbon nanotubes (MWCNT) as a bio-lubricant for compression ignition engines. MWCNT, dispersed in EJA using an ultrasonic probe sonicator with Triton X-100 as a surfactant, was tested at nanoparticle concentrations from 0.5 to 2 wt%. Engine performance and emission characteristics were assessed using SAE 20W40, EJA, and EJA-MWCNT in a four-stroke diesel engine. Results showed that EJA with 2 wt% MWCNT reduced friction power by 39.13% compared to SAE 20W40 and decreased brake specific energy consumption by 6.11%, while lowering emissions of hydrocarbons, carbon monoxide, and smoke. These findings highlight EJA-MWCNT as a promising lubricant for diesel engines, enhancing efficiency and reducing pollutants, making it a viable eco-friendly substitute for diminishing petroleum resources.

## Introduction

Globally, numerous initiatives have been made to increase the automobile industry’s dependability, energy efficiency, and environmental friendliness. The use of lightweight materials treated exhaust gas, less hazardous fuel, and controlled combustion are just a few examples of technological advancements that can help reduce the environmental problems that machines and vehicles cause^1^. To minimize wear and friction, especially in engines and drive trains, an automobile vehicle must have adequate lubrication at the appropriate operating conditions for safe and dependable operation. Mineral oil has been a lubricant for automotive applications for a very long time. However, the usage of mineral oil is restricted to the availability of crude oil reserves because it is the byproduct of crude oil’s distillation. Furthermore, the dumping of mineral oil results in the deterioration of ecosystems on land and in water due to its disposal^2^. In addition, the release of metal traces such as calcium, magnesium, iron particles, and zinc because of burning mineral oil for lubrication raises concerns about environmental deterioration^3^. Furthermore, it has been projected that mineral oil’s future as a lubricant in car engines will be dreadful^4^. Finding a viable substitute for mineral oils in internal combustion engines is therefore necessary. A creative worldwide search has begun to create and use alternative lubricants to safeguard the environment from the damage caused by lubricating oils and their uncontrolled spills, as a result of the depletion of crude oil reserves and the increase in oil costs^5^.

A lubricant made of chemical ingredients that have been artificially manufactured is called synthetic oil. Instead of using crude oil, synthetic lubricants can be manufactured using chemically altered petroleum components. Crude oil is the most widely used starting material; it is separated and subsequently undergoes physical and chemical transformations. When it comes to performance, synthetic lubricants can outperform conventional mineral-based oils^6^. They have a longer service life, which promotes environmental sustainability, because of their reduced volatility and greater thermal stability. Compared to mineral lubricants, synthetic lubricants offer several benefits, including as improved thermal oxidation, and low-temperature fluidity. It serves as an alternative to refined petroleum-based oils in situations involving extremely high or low temperatures. Synthetic oils are used in metal stamping in place of conventional lubricants made of petroleum and animal fat because they offer advantages for the environment and other factors^7^. Apart from the benefits, a few drawbacks have also restricted their application as lubricants. The more expensive nature of synthetic oil is perhaps its biggest drawback. The cost of synthetic oil is two to four times higher than that of conventional oil. For synthetic oils, cold storage may raise the possibility of additive precipitation^8,9^. Additionally, the disposal of synthetic oils can pose a serious threat to ecosystems.

In comparison to mineral oils, bio-lubricants derived from vegetable oils have more lubricity, a higher flash point, less volatility, and a higher viscosity index. Vegetable oils are suited for boundary and hydrodynamic lubrication because of their long-chain fatty acid and polar group composition^10^. A variety of sustainable feedstocks can be used to make bio-lubricants^11^. Edible vegetable oil feedstocks have constraints in terms of suitability because of contemporary issues such as their role in the human food chain and the environmental harm that results from using available cultivation land. The usage of edible vegetable oil feedstocks may result in ecological imbalances since plantations require the clearing of forest area^12^. Hence, non-edible plant oils could be widely used as lubricants including in the Internal combustion engines^13^.

Studies were evaluated by Bekal and Bhat^14^ on diesel engines by using mineral oil and pongamia oil as lubricants. Their studies revealed that usage of pongamia oil lubricant at medium and high load situations resulted in the lowest brake specific energy consumption (BSEC) and the highest brake thermal efficiency (BTE). As pongamia oil does not contain any metal elements, unlike mineral oil lubricants, its use as a lubricant could increase efficiency and fully remove the emission of metal traces. Kumar and Dinesha^15^ also evaluated the studies on diesel engines using gingelly oil (sesame oil) as a bio-lubricant and found that esterified gingelly oil had the highest BTE and lowest brake-specific fuel consumption (BSFC) when compared with neat gingelly and mineral oil. Gingelly bio-lubricant reduced engine pollutants, including NOx, CO, and smoke emissions, when compared to mineral oil as a lubricant. To improve engine performance, it is crucial to minimize the boundary friction coefficient in the piston ring assembly, as the frictional power losses at the piston ring/cylinder liner contacts range from 35 to 45%^16,17^. The goal of the study done by Gulzar et al.^18^ was to examine the nanoparticles as potential nano lubricant additives to enhance engine tribological performance, oil quality, and fuel efficiency. The tribological characteristics of engine oils were significantly impacted by the nanoparticles. Since they are incredibly small and have high specific surface areas, nanoparticles vary from conventional bulk materials^19^.

Due to their employment in tribological applications as solid lubricants on rubbing surfaces, anti-wear additives, and friction modifiers, nano-lubricant additives have drawn special attention^20^. Since the nanoparticles have superior tribological qualities and increased load-carrying capacity, they have a special significance in nano-lubrication^21^. Studies were conducted by Sarma et al.^22^ on the Racer-4 lubricant from Hindustan Petroleum Corporation (India) that was distributed with varying mass concentrations of Cu and TiO_2_ nanoparticles. In terms of the operation of an engine with Racer-4 of HPCL, the addition of nano Cu in mass fractions of 0.05%, 0.1%, and 0.2% to nano lubricants resulted in improved brake thermal efficiencies. Results were found to be superior with a 0.1% nano Cu-dispersed lubricant than with mass concentrations of 0.05 and 0.2%. Numerous research studies have been carried out on the Racer-4 lubricant manufactured by Hindustan Petroleum Corporation in India.

In summary, adding nanoparticles to a synthetic base oil improves its anti-friction properties while lowering wear. Extensive research has been conducted on the combination of synthetic or mineral oil with nanoadditives. There is limited research on adding nanoparticles to vegetable oil-based bio-lubricants. The outcome of adding nanoparticles to bio-lubricants is affected by various factors, such as the type of oil, the type of nanoparticle, its concentration, and its size. In order to fully understand the benefits of adding nanoparticles, more research is required to examine their physiochemical and rheological characteristics, the agglomeration and concentration of nanoparticles, their ability to vary in volume and size, and other related factors. To address the above research gaps, In this study, authors have chosen Jatropha-based bio-lubricant as a non-edible bio-lubricant. Various volume concentrations of MWCNT nanoparticles, procured from a local vendor with an outer diameter of 10–20 nm and length of 5–10 μm, were added to jatropha bio-lubricant to evaluate their impact on engine performance. The study included adding MWCNT nanoparticles at 0.5, 1, 1.5, and 2 wt% to the bio lubricant, followed by an analysis of the engine’s performance and emission test.

## Methodology

### Preparation of bio-lubricant

Vegetable oils have a lot of potential as bio-lubricants, but due to their high heterogeneity and other undesirable physical characteristics—like low temperature properties and poor oxidation stability—they are not frequently commercialized. The two most widely used processes for altering vegetable oils are transesterification and epoxidation. An ester’s organic group reacts with the organic group of its alcohol to produce a transesterification process. The reaction is frequently aided by the use of an acid or base catalyst. Epoxidation is the process by which hydrogen peroxide, organic peroxides, etc. transform the carbon–carbon double bond into oxiranes (epoxides)^23^.

### Transesterification and epoxidation of vegetable oil

**Fig. 1:**
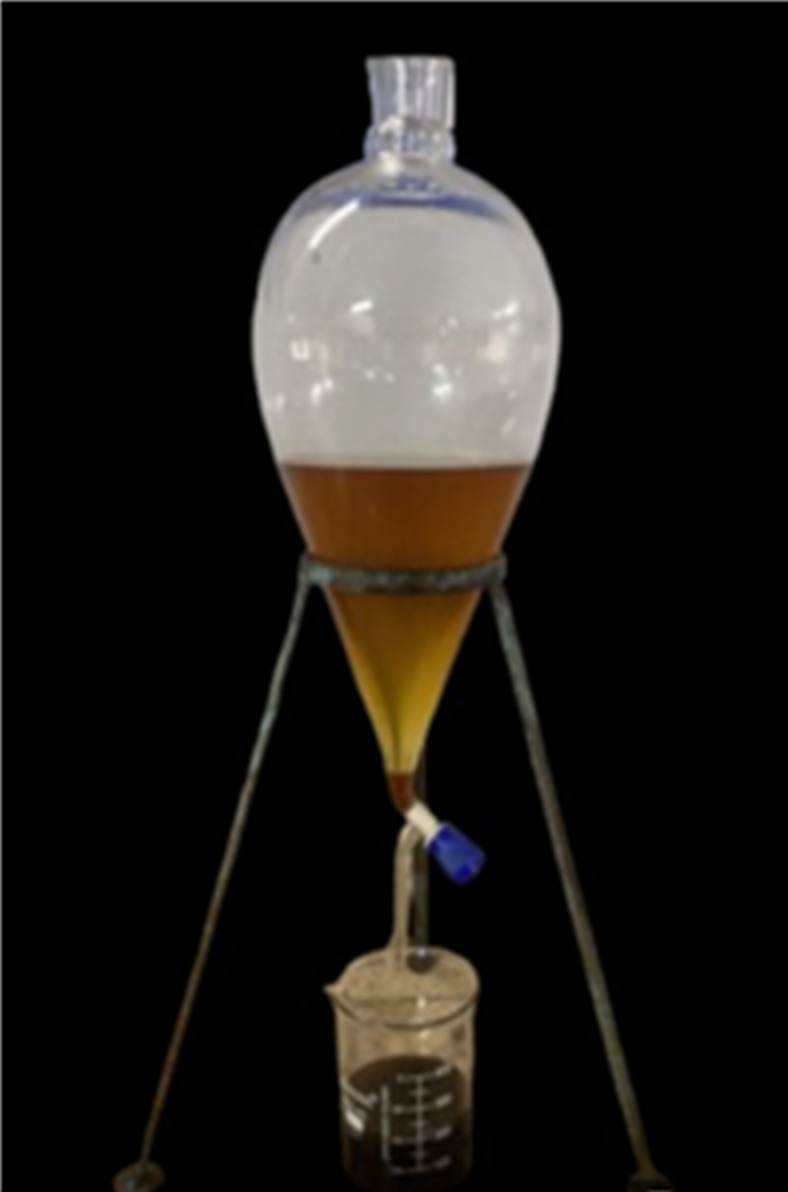
Separation of glycerol and methyl ester.

**Fig. 2:**
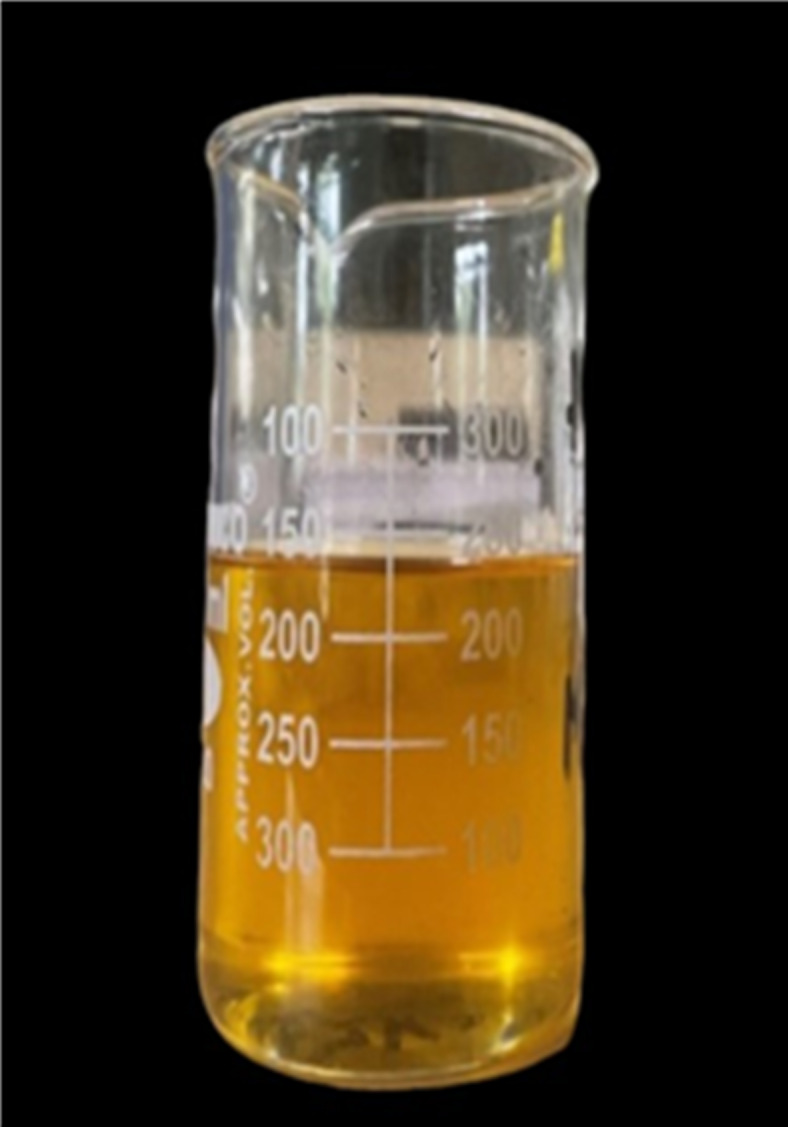
Final epoxidized product.

A solution containing methanol and sodium hydroxide is added to a three-neck flask containing 250 ml of jatropha raw oil heated at 600 rpm with magnetic stirring. Heating is done for duration of 120 min and further prepared sample is kept in separating flask for separation of methyl ester and glycerol as depicted in Fig. 1. Further, methyl ester was washed with warm and cold water, the transesterified oil was placed in a beaker to undergo epoxidation. After being mixed for 30 min at room temperature, a combination of transesterified jatropha, peracetic acid, and H_2_SO_4_ catalyst was stirred magnetically. After adding hydrogen peroxide to the mixture dropwise, the mixture was heated for 3 h. The mixture was supplemented with diethyl ether and stored in a separating flask. Subsequently, the flask’s bottom developed an aqueous layer, which was emptied later. After that, warm and cold water were used to cleanse the solution. The solution was then treated with anhydrous sodium sulphate to remove the water content. The epoxidized solution was then filtered and kept in a beaker for later use as shown in Fig. 2.

### Characterization of the bio-lubricant sample with nanoparticle additive

A Jatropha bio-lubricant sample was prepared using an H_2_SO_4_ catalyst. Multiwalled carbon nanotubes (MWCNT) were used in the present study. To prevent agglomeration, surfactants are added to the mixture of nanoparticles and bio-lubricants to increase stability. Surfactant modification is one of the most widely used methods for nanoparticle dispersion. Molecules that spontaneously bond with each other in forming sealed bubbles are known as surfactants. Triton X-100 is used as a surfactant for our study. The MWCNT nanoparticles of 1 wt% were added to the prepared jatropha bio-lubricant and sonicated in an ultrasonic probe sonicator. After sonication with an ultrasonic probe for 30 min, nanoparticles were dispersed well in bio-lubricants. A comparative analysis of the properties of SAE 20W40, bio-lubricants, and their nanoparticle additions is shown in Table 1.

**Table 1:** Comparative study of the properties of the bio-lubricants and their nanoparticle additives along with SAE20W40.

Physicochemical properties	Instrument	SAE 20W40	Jatropha bio-lubricant	Jatropha bio-lubricant with MWCNT additive (2 wt%)
Density (kg/m^3^)	Hydrometer	902	910	940
Flash point (ºC)	Cleveland Open-Cup	210	225	265
Fire point (ºC)	Cleveland Open-Cup	223	238	275
Pour point (ºC)	Ducom Pour Point Tester	+ 7	− 8	− 5
Thermal Stability (ºC)	Perkin Elmer’s TGA-400	190	238	270
Viscosity at 80 ºC (mPa s)	Rheometer	21.6	5.1	9.9
Viscosity index	Rheometer	110	180	240

## Experiment test rig

**Fig. 3:**
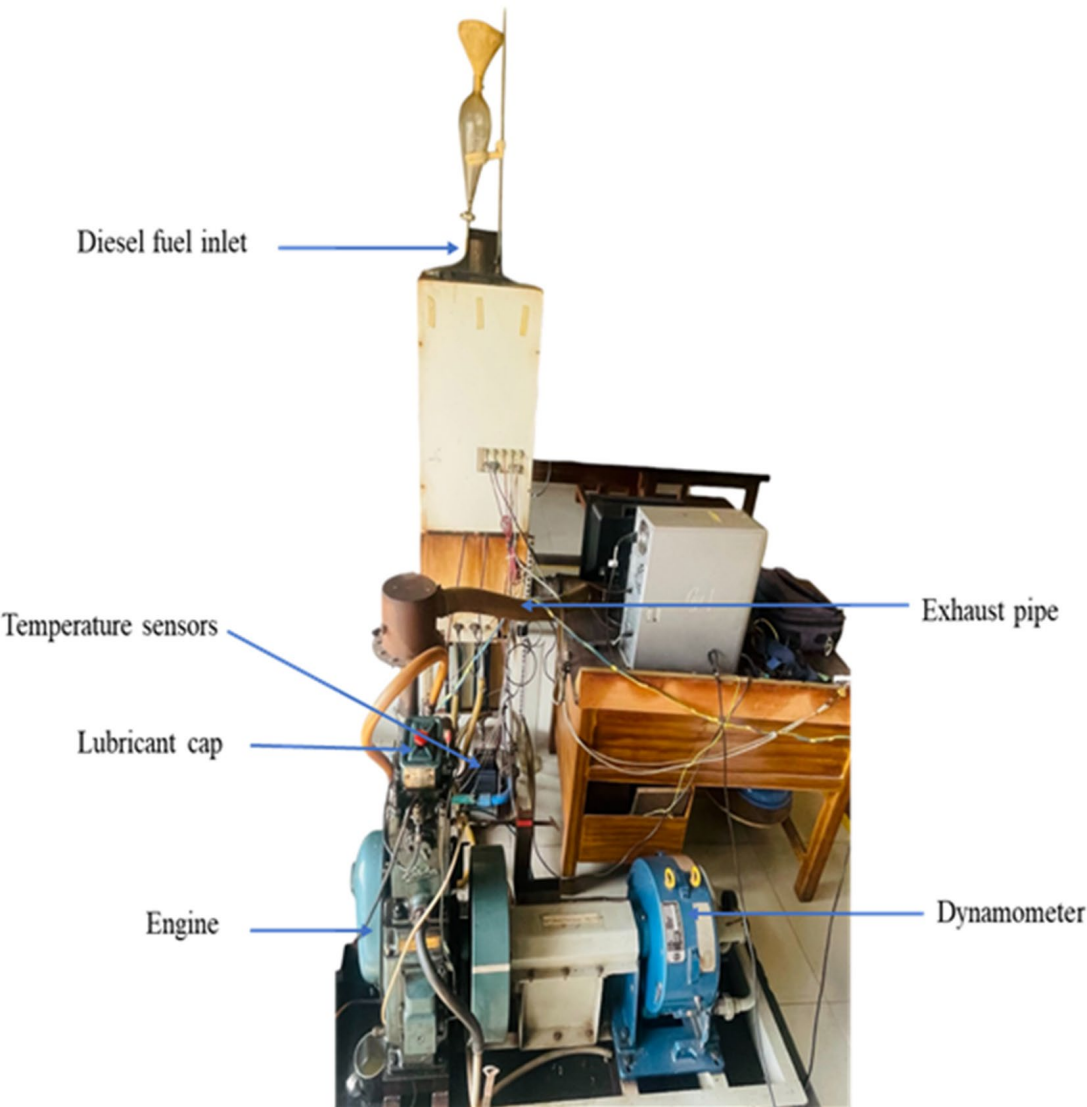
Experimental setup of diesel engine.

The study involved characterizing bio-lubricant samples enhanced with nanoparticle additives and conducting experiments to assess their effects on diesel engine performance and emissions. Comprehensive experimental testing was carried out on a single-cylinder, four-stroke diesel engine using diesel as the primary fuel. The experimental setup, depicted in Fig. 3, included an eddy current dynamometer for engine loading, temperature sensors at critical points for precise measurements, and a rotameter to monitor coolant and jacket water flow rates. A burette was connected to the fuel line for accurate determination of fuel flow rates. Detailed engine specifications are outlined in Table 2, while the measurement ranges and specifications of the instrumentation are summarized in Table 3.

Initially, performance and emission tests were conducted using diesel fuel with SAE 20W40 lubricant (in crankcase) as a baseline. Subsequently, the engine was tested with bio-lubricants such as EJA and EJA blended with multi-walled carbon nanotubes (EJA-MWCNT) to evaluate their influence on engine performance parameters and emission characteristics.

The following combinations of lubricants / bio-lubricants with diesel fuel are used for experimentation.

- Diesel + SAE 20W40.
- Diesel + EJA.
- Diesel + EJA-MWCNT (volume fraction varied from 0.5 to 2% with an increment of 0.5 each time).

For each test instance, the engine load was incrementally increased from zero to maximum in steps of 25%. During these tests, the time required for the consumption of 10 cc of fuel was recorded, along with data on fuel consumption rate, engine load, and exhaust gas temperature. Emissions were analyzed using a five-gas analyzer to measure NO_x_, CO, CO₂, and HC, while an AVL smoke meter was employed to assess smoke opacity. Key performance parameters and emission characteristics were calculated using standardized equations. The results were then compared across all test scenarios, evaluating the impact of nanoparticle-enhanced bio-lubricants versus conventional lubricants on engine performance and emissions.

**Table 2:** Engine specifications.

Specification	Value or description
Type	Single cylinder, four stroke, air cooled engine
Bore x stroke	87.5 × 110 mm
Compression ratio	17.5:1
Rated output	5.2 kW at 1500 rpm
Injection pressure	180 bar
Swept volume	661 cm^3^

**Table 3:** Range and resolution of instruments.

Item	Range	Resolution
Pressure	0.344–75 bar	0.0069 bar
Smoke opacity	0–100	0.01
NO_x_ concentration	0–5000 ppm	1 ppm
CO concentration	0–10% vol	0.01% vol
HC concentration	0–20,000 ppm	10 ppm
CO_2_ concentration	0–20% vol	0.1% vol

### Uncertainty analysis

The uncertainties in the relevant factors are quantified by uncertainty analysis. All measurements include an error component, although we usually try to keep them as little as practical. However, we require an accurate approximation of their dimension. The total uncertainty of a response can be computed as follows when it is influenced by multiple causes and the uncertainties in the independent variables are all equally probable:

1 $${\text{W}}_{{\text{R}}} = \pm \sqrt {\left( {\frac{{\partial {\text{R}}}}{{\partial {\text{X}}_{1} }}} \right)^{2} \times \left( {{\text{W}}_{1} } \right)^{2} + \left( {\frac{{\partial {\text{R}}}}{{\partial {\text{X}}_{2} }}} \right)^{2} \times \left( {{\text{W}}_{2} } \right)^{2} + \cdots + \left( {\frac{{\partial {\text{R}}}}{{\partial {\text{X}}_{n} }}} \right)^{2} \times \left( {{\text{W}}_{{\text{n}}} } \right)^{2} }$$

For a specific operating situation, the uncertainties in computed values such fuel and air mass flow rates, brake specific energy consumption, braking power, and brake thermal efficiency are determined, using Eq. (1) by the approach of Kline^24^. By using the percentage uncertainty for independent parameters, the uncertainty of the dependent parameters has been found and is shown in Table 4.

**Table 4:** Uncertainties in the parameters and instruments used in experiments.

Measurement items	Percentage uncertainty
Air flow rate	1.2
Load	0.65
Speed	0.51
Fuel flow rate	0.8
Brake thermal efficiency	1.1
Brake specific energy consumption	1.3
HC emission	2.1
NO emission	2.0
Smoke emission	0.65
CO emission	1.42
CO_2_ emission	0.72

## Results and discussions

### Performance test

Performance parameters for the diesel engine using SAE 20W40 lubricant and bio-lubricants such as EJA and EJA-MWCNT at various loads are calculated. FP (kW) is calculated by using Willian’s line method by plotting the variation of TFC with BP. Figure 4 shows friction power obtained for SAE 20W40, EJA and EJA + 2 wt% MWCNT.

#### Friction power

**Fig. 4:**
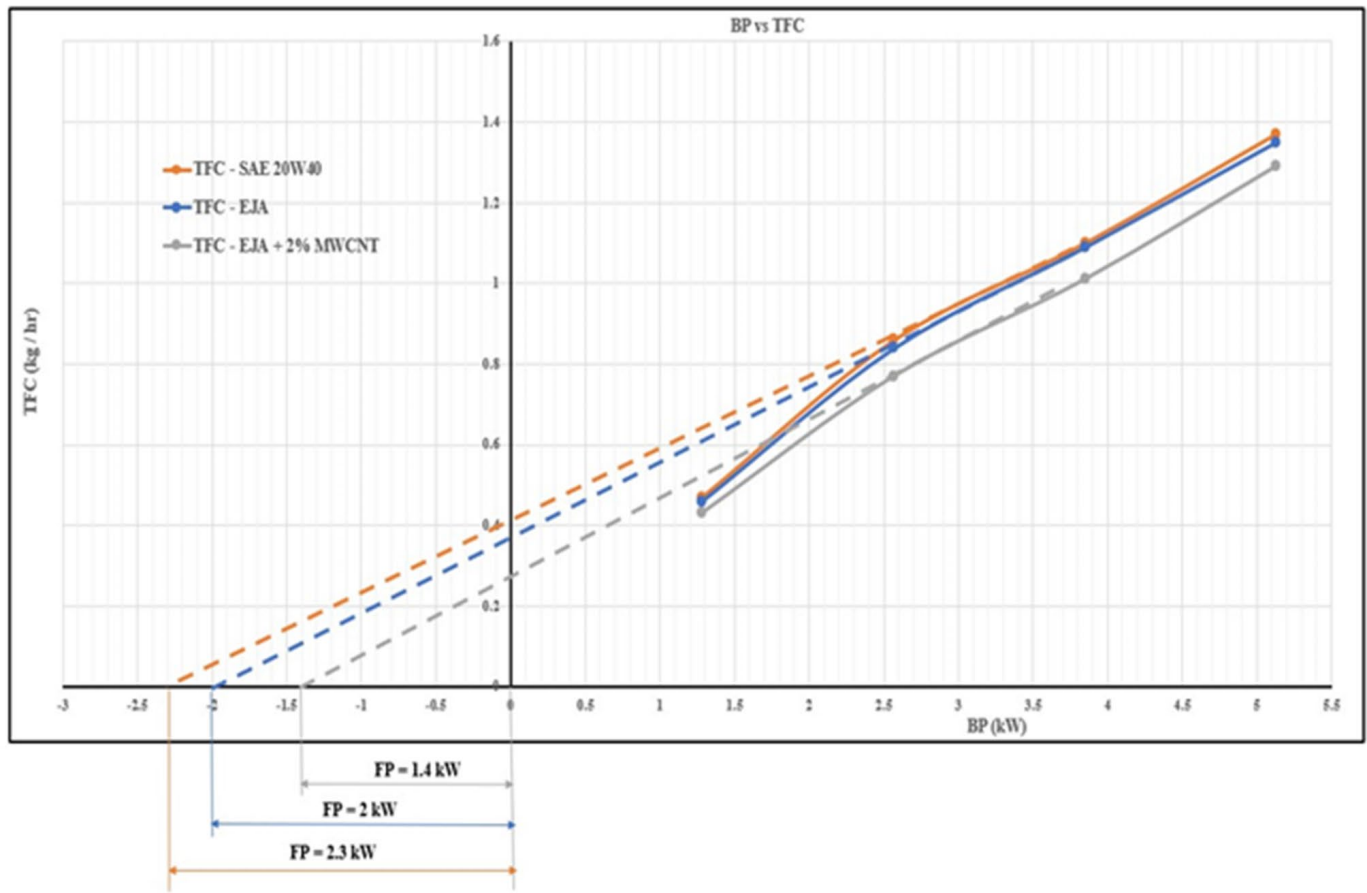
Friction power for SAE 20W40, EJA and EJA + 2 wt% MWCNT.

### Engine’s performance

It illustrates the plotting of total fuel consumption against brake power for 3 samples - SAE 20W40, EJA, and EJA + 2 wt% MWCNT. EJA has less friction power than SAE 20W40 due to the exceptional qualities of EJA bio-lubricants. With greater oxidation resistance and the ability to create a superior protective barrier, even small changes can make a significant impact on efficiency and performance. Avoiding deposits and sludge build-up is crucial for reducing friction. EJA’s oxidation resistance is due to natural antioxidants, ensuring engines run smoothly and efficiently. The addition of MWCNT nanoparticles to bio-lubricants enhances the lubrication properties, reducing frictional losses in the engine. Improved lubrication contributes to lower mechanical losses and, consequently, reduced BSEC. Another reason for the reduction in friction power is – MWCNT additives can act as lubricating additives, forming a protective layer on the surfaces of moving engine components. This layer helps reduce direct metal-to-metal contact and minimizes friction between surfaces, leading to lower friction power losses. The addition of MWCNT nanoparticles to EJA bio-lubricant causes reduces ignition delay and the combustion starts sooner. The addition of nanoparticles will replace the sliding contact between the piston and cylinder body with a point contact or line contact. Also, nanoparticles having higher thermal conductivity help to effectively remove the heat generated at the interface to the cooling jacket, then limiting the temperature rise at the interface of the piston and cylinder body. These factors help in reducing the FP of the engine which leads to an increase in the engine performance. The percentage reduction in friction power for EJA + 2 wt% MWCNT bio-lubricant was found to be 39.13% compared to SAE 20W40.

#### Brake thermal efficiency

**Fig. 5:**
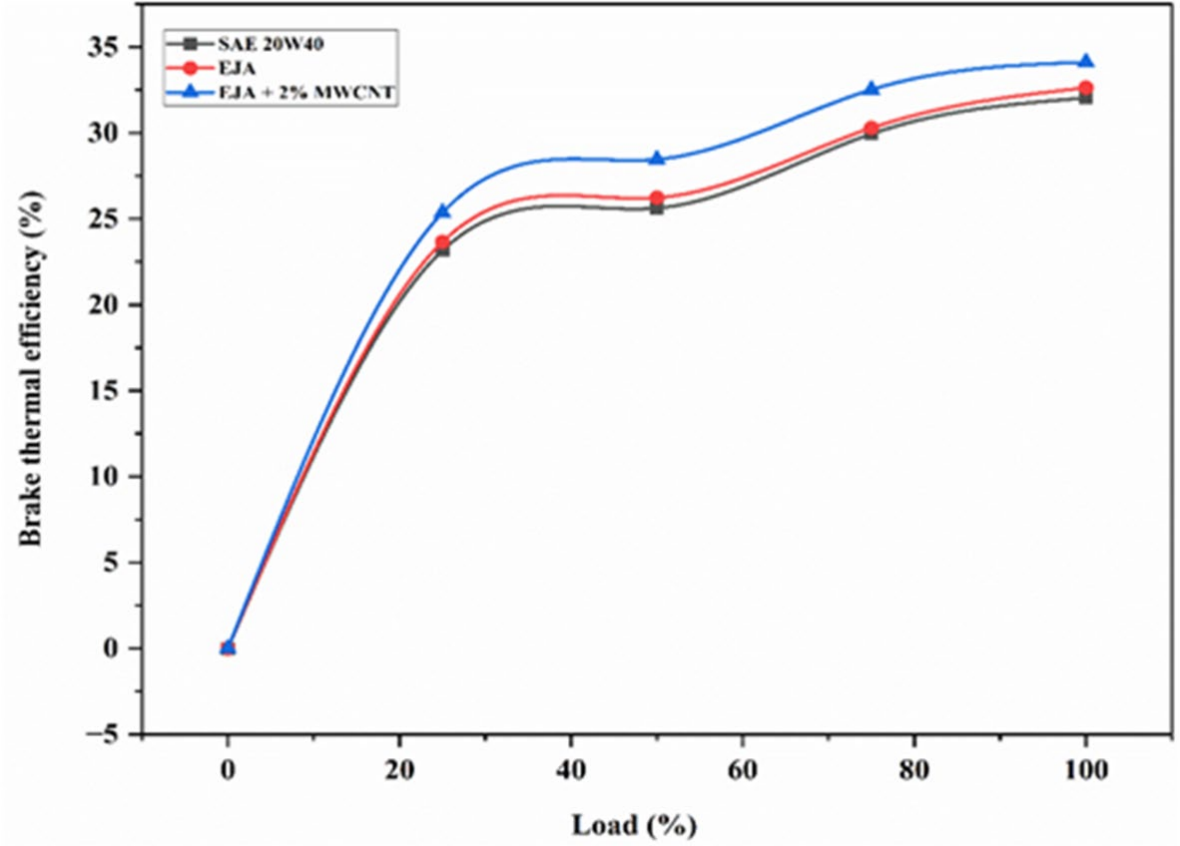
Variation of BTE for SAE 20W40, EJA and EJA + 2 wt% MWCNT nanoparticle concentrations.

Figure 5 illustrates brake thermal efficiency variation against various loads for 3 samples - SAE 20W40, EJA, and EJA + 2 wt% MWCNT. Brake thermal efficiency (BTE) is defined as the ratio of the observed brake power to the product of the fuel flow rate and its calorific value. It is evident that the vegetable-based lubricant i.e. EJA has a higher brake thermal efficiency than SAE 20W40 lubricant due to the increased availability of oxygen, which essentially boosts the fuel’s ability to convert thermal energy into work. High flash point, high viscosity index and good resistance to shear make bio-lubricant better than SAE 20W40 lubricant. Improved lubricity of the bio-lubricant due to altered chemical characteristics during epoxidation results in optimal lubrication of the engine body. Enhanced lubricity and more oxygen in its molecular structure, both of which support efficient burning.

During engine operation, although the lubricant is typically confined to the engine’s moving parts and not intended to mix with the fuel in the combustion chamber, there is a possibility that small amounts of lubricating oil may seep into the combustion chamber during the engine’s reciprocating motion at specific RPMs. Over time, wear in piston rings or valve seals can progressively worse, especially under conditions of high load and pressure. This increased wear can lead to a flow of lubricant into the combustion chamber, where it mixes with the fuel-air mixture, potentially impacting combustion performance.

EJA bio-lubricant, naturally contain oxygen molecules within their chemical structure, in the form of esters, fatty acids, and glycerol molecules. When EJA bio-lubricant is used in an internal combustion engine, it can participate in the combustion process, providing additional oxygen. Although most oxygen for combustion comes from the air in the intake system, the oxygenated molecules in the bio-lubricant contribute to a more complete combustion of the fuel as depicted in the graph. Further addition of MWCNT nanoparticle additive to the EJA helps in the increase of BTE as the high surface area-to-volume ratio of nanoparticles improves their capacity for heat transfer. MWCNT helped in increasing the effectiveness of heat transfer between engine components when added to EJA. If any lubricant with nano additives leaks into the combustion chamber, it can enhance heat transfer due to its properties, such as higher surface-to-volume ratio and improved thermal conductivity. This can reduce ignition delay and cause combustion to start a bit early. As a result, efficient combustion takes place leading to higher heat release rate. This enhances overall thermal management by lowering the chance of overheating and helping to dissipate excess heat more efficiently. Hence, EJA + 2 wt% MWCNT having highest brake thermal efficiency than EJA and SAE 20W40. The percentage increase in BTE for EJA + 2 wt% MWCNT is 4.56% and 6.49% as compared to EJA and SAE 20W40 respectively. Therefore, MWCNT additive to bio-lubricant may be utilized to increase heavy-duty diesel engines’ efficiency.

**Fig. 6:**
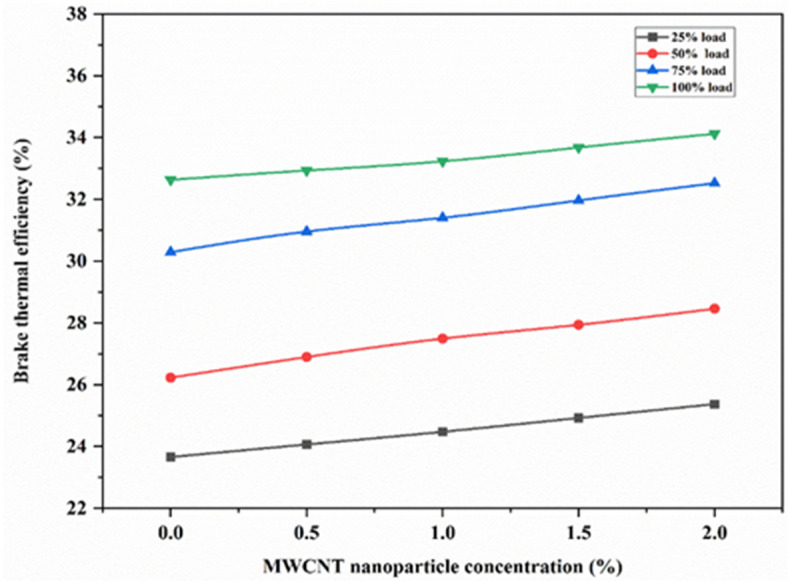
Variation of BTE for various MWCNT nanoparticle concentration.

Figure 6 illustrates plotting of BTE against various concentrations of MWCNT for loads ranging from 25 to 100%. As MWCNT concentration increases, BTE increases with increasing load. The 2 wt% MWCNT exhibited higher thermal conductivity than the remaining concentrations, by facilitating better heat distribution and dissipation, preventing localized hotspots within the engine. Increasing concentrations from 0.5 to 2 wt% helped in the improvement of thermal conductivity, which consequently led to more uniform temperatures across the engine, promoting efficient combustion with an increase in BTE and reduction in friction power. Increasing nanoparticle concentration can improve engine performance by raising Brake Thermal Efficiency (BTE) due to higher heat release rates and reduced friction power. The percentage increase in BTE for full load at 2 wt% MWCNT was found to be 4.56% as compared to full load for EJA without nanoparticle additive.

#### Brake specific energy consumption

**Fig. 7:**
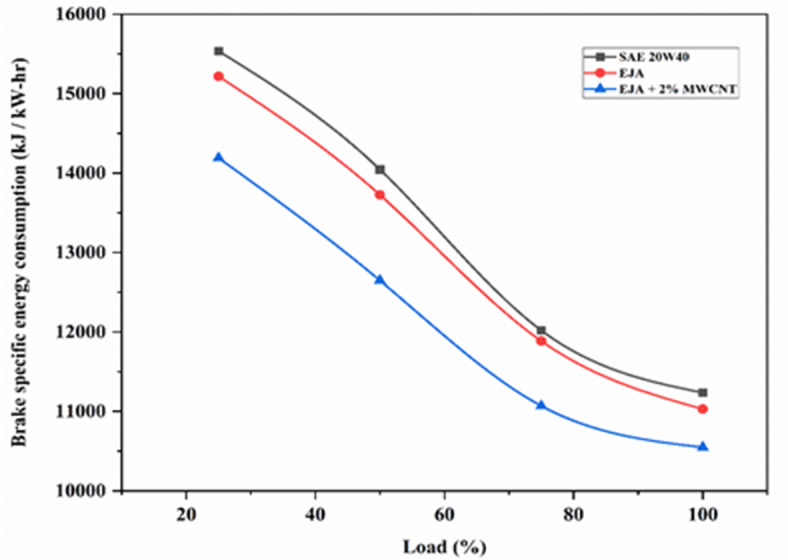
Variation of BSEC for SAE 20W40, EJA and EJA + 2 wt% MWCNT nanoparticle concentrations.

**Fig. 8:**
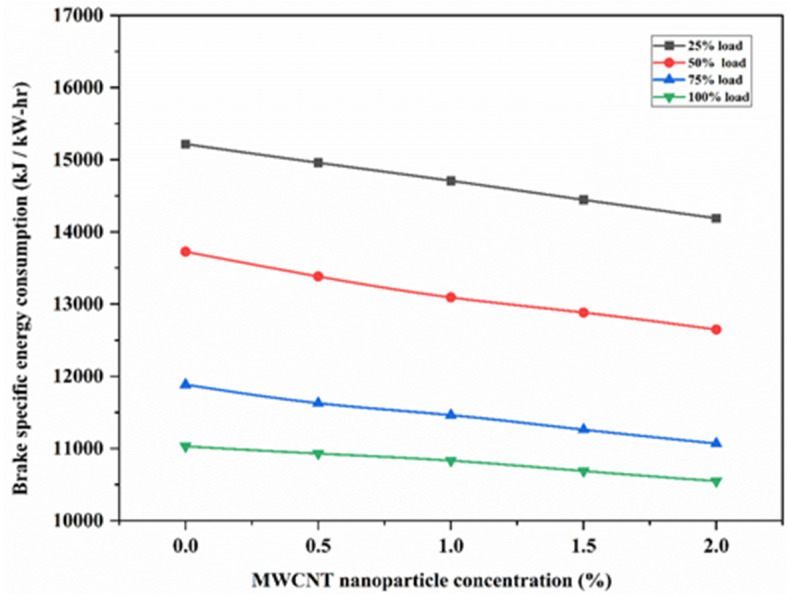
Variation of BSEC for various MWCNT nanoparticle concentrations.

Figure 7 illustrates the plotting of the brake-specific energy consumption (BSEC) against various loads for 3 samples - SAE 20W40, EJA, and EJA + 2 wt% MWCNT. It is evident from Fig. 8 that an increase in load leads to a decrease in BSEC. This is mainly because engines tend to run closer to their ideal operating conditions under increasing loads, which improves combustion efficiency. This indicates that a higher proportion of the fuel burns completely, releasing more energy from the fuel for a given quantity. The bio-lubricant EJA has the lowest BSEC compared to SAE 20W40 lubricant under all loading conditions. The reason is that triglycerides, primarily a polar group of ester molecules in nature, are found in bio-lubricants. The triglyceride chain improves the frictional properties and lubricating film strength. Improved lubricating qualities of vegetable-based lubricants lower frictional losses, which aid in the fuel’s full combustion. Adding MWCNT to EJA bio-lubricant lowers the amount of fuel used in an engine, since it oxidizes carbon deposits from the engine, resulting in smooth and efficient operation resulting in less BSEC. EJA + 2 wt% MWCT bio-lubricant leads to a reduction in BSEC as MWCNT additives lead to a shorter ignition delay period and slightly greater heat release rates during portions of the expansion stroke, which improve power and specific fuel consumption values. Another factor for reduction in BSEC for EJA + 2 wt% MWCNT bio-lubricant is - Improved heat transfer properties of MWCNT nanoparticles, which resulted in reducing the need for excessive cooling, allowing for more efficient operation and reducing the energy consumed by cooling systems. The percentage reduction in BSEC for EJA + 2 wt% MWCNT bio-lubricant was found to be 4.36% and 6.11% compared to EJA and SAE 20W40 respectively.

Figure 8 illustrates the plotting of BSEC against various concentrations of MWCNT for loads ranging from 25 to 100%. As MWCNT concentration increases, BSEC decreases with increasing load. This is because increased loads frequently result in higher pressure and temperature inside the combustion chamber, which can lessen heat loss to the environment. An increased percentage of fuel combustion energy turned to productive work is a result of improved heat retention. With the increase in MWCNT nanoparticle additive, the reduction in BSEC was higher because the higher concentration of MWCNT exhibits higher catalytic properties leading to better combustion efficiency. As 2 wt% MWCNT at full load promotes more complete combustion of the fuel-air mixture by contributing to increased power generation per unit of fuel, leading to lower BSEC. The percentage reduction in BSEC for EJA + 2 wt% MWCNT at full load was found to be 4.36% compared to bio-lubricant EJA without nanoparticle additive.

#### Temperature exhaust

**Fig. 9:**
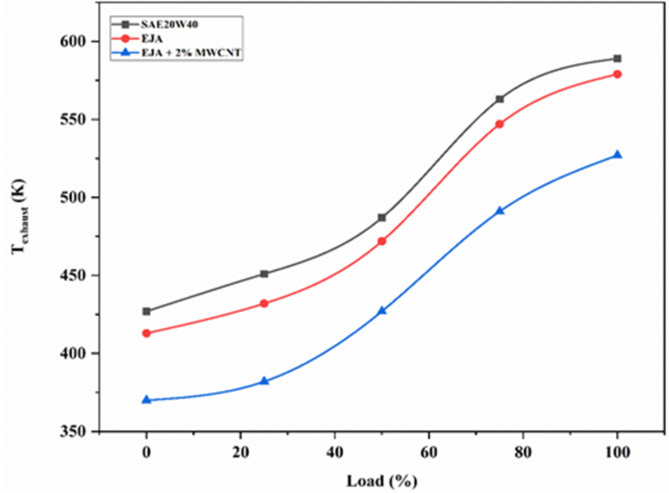
Variation of T_exhaust_ for SAE 20W40, EJA and EJA + 2 wt% MWCNT nanoparticle concentrations.

**Fig. 10:**
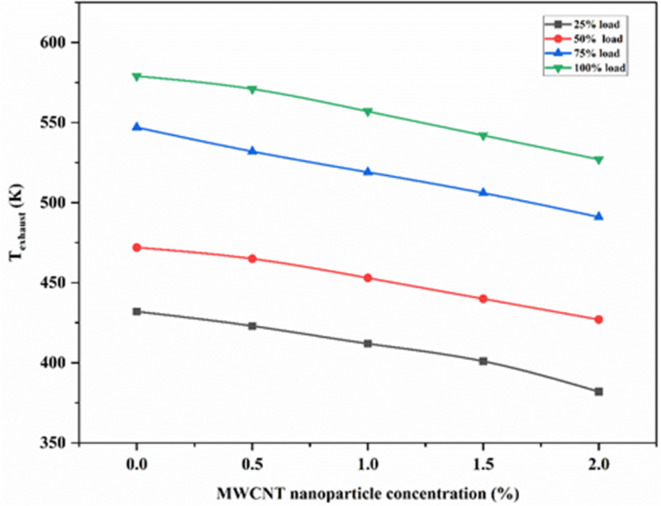
Variation of T_exhaust_ for various MWCNT nanoparticle concentrations.

It is observed from Fig. 9 that the exhaust gas temperature (T_exhaust_) was found to be less for EJA than SAE 20W40 as bio-lubricants contain a polar group of ester molecules resulting in complete combustion with less frictional losses causing less T_exhaust_. It can be also seen from Fig. 10 that the exhaust gas temperature (T_exhaust_) was found to be less for EJA + 2 wt% MWCNT bio-lubricant compared to that of EJA and SAE 20W40 at higher loading conditions. As the loading condition increases, the T_exhaust_ also increases because more combustion occurs per unit of time when the engine is operating at a higher load. After all, it is burning a greater volume of air-fuel combination. Higher temperatures in the combustion chamber as a result of the enhanced combustion activity raise the temperatures of the exhaust gases. Due to complete combustion occurring with EJA + 2 wt% MWCNT bio-lubricant, T_exhaust_ is lowest compared to EJA and SAE 20W40. Due to the addition of EJA bio-lubricant and nanoparticles, the ignition delay may be slightly reduced, resulting in early combustion. This, in turn, ensures a more complete combustion with reduced after-burning. Furthermore, if any lubricant is present in the combustion chamber, it can easily mix with the diesel sample and ensure a more complete combustion than when mixed with SAE. Additionally, the bio-lubricant also reduces the frictional power. These features led to the complete combustion of fuel during both controlled and uncontrolled phases releasing more heat with decreasing after burning. This results in lowering the exhaust temperature. When the MWNT nanoparticles have been mixed with the bio-lubricant heat transfer rate at the interface of reciprocating parts will be judiciously increased. It also leads to the reduction of exhaust gas temperature. 10.52% was the percentage reduction for bio-lubricant EJA + 2 wt% MWCNT as compared to SAE 20W40.

Figure 10, depicts the variation of T_exhaust_ against various concentrations of MWCNT for loads ranging from 25 to 100%. As the nanoparticle concentration increases, T_exhaust_ decreases because during combustion, MWCNT nanoparticles have an impact on the stability and properties of the flame front. This has an effect on the temperatures of the combustion gases and, in turn, the exhaust gases. As the concentration of nanoparticles increases, a larger quantity of nanoparticles will interact with the lubricant, resulting in a better heat transfer rate during the reciprocating motion of the piston. At the same time higher concentration will also improve the combustion performance and collectively reducing the exhaust gas temperature. EJA + 2 wt% MWCNT has lowest T_exhaust_ and there was 8.98% reduction compared to EJA without nanoparticles.

### Combustion characteristics

**Fig. 11:**
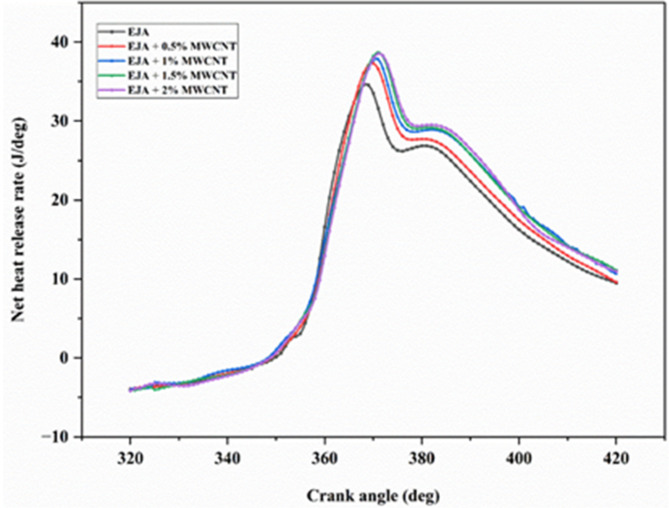
Variation of Net heat release rate for various MWCNT nanoparticle concentrations.

Figure 11 depicts the variation of the net heat release rate with crank angle for the engine operation at different lubricant combinations. The bio-based lubricant has enhanced lubricity and inbuilt oxygen content and promotes more complete burning when it covers the combustion chamber surface. The addition of MWCNT nanoparticles to EJA enhances heat transfer due to a higher surface-to-volume ratio and improved thermal conductivity. It transfers heat between the nearby fuel droplets and causes rapid combustion by reduced ignition delay. The heat release rate (HRR) of MWCNT mixed EJA lubricants is higher than the neat EJA lubricant operation. An increase in the percentage composition of MWCNT in EJA exhibits an increase in HRR due to increased combustion quality. This has also led to an increase in useful power output and a reduction in engine pollutants. Among the EJA + MWCNT lubricant combinations, EJA + 2 wt% MWCNT resulted in maximum HRR and is 3.24% as compared with EJA + 0.5 wt% MWCNT bio-lubricant operation.

#### Emission test

The emission parameters for SAE 20W40, and EJA with various nanoparticle concentrations of MWCNT have been measured, compared, and discussed below.

#### CO emissions

**Fig. 12:**
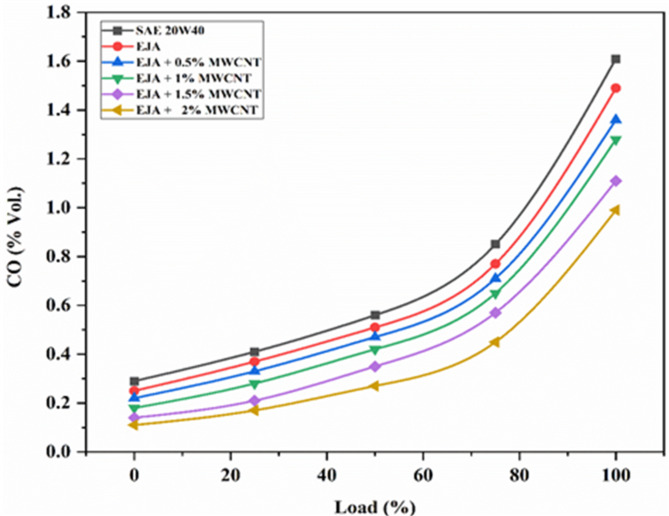
Variation of CO for SAE 20W40 and EJA with various MWCNT nanoparticle concentrations.

The primary byproduct of complete carbon combustion, or when there is sufficient air present, is carbon dioxide (CO_2_). CO is created when there is insufficient air available to complete the burning of carbon. Lack of oxygen for the oxidation reaction is the primary cause of CO production. The entire conversion of CO to CO_2_ can be difficult when the carbon activation temperature is high, as it is during the combustion of a typical diesel engine. The conversion process also takes longer because of the greater activation temperature. By adding MWCNT to the bio-lubricant and thus lowering the carbon activation temperature. So, CO can oxidize to CO_2_ at low temperatures. This event may result in a more thorough conversion of CO to CO_2_, which would lower CO emissions. From Fig. 12 it is evident that for all tested lubricants, CO emissions are extremely low at low loads and exponentially increase after 50% load. The SAE 20W40 lubricant exhibited a significant proportion of CO emission due to the incomplete combustion, while the EJA and EJA + 2 wt% MWCNT bio-lubricant displayed the least amount of CO emission because bio-lubricant have additional oxygen content which helps in having complete combustion. Higher loads are favored by the bio-lubricant’s excess oxygen. As a result, CO emissions are decreased. The percentage reduction in CO emission for full load for EJA + 2 wt% MWCNT is 33.55% and 38.50% as compared to EJA and SAE 20W40 respectively. When jatropha is used as the lubricant, the extra oxygen present in their molecular structures will satisfy the oxygen requirements, and hence, more CO gets converted into CO_2_ whenever the lubricant comes in contact with the fuel at the combustion chamber or nearby zone. On the other hand, combustion using mineral oil lubricants possesses oxygen starvation and this will lead to incomplete combustion and hence more CO at the engine exhaust. Further addition of nanoparticles will effectively manage the thermal conditions inside the combustion chamber due to their higher thermal conductivity and higher surface-to-volume ratio. Ignition delay is reduced, and combustion starts a little early, leading to complete combustion and releasing maximum heat. Hence CO in the exhaust will be reduced or converted into CO_2_. When the nanoparticle concentration is increased from 0.5 to 2% CO emissions were reduced by 27.20%.

#### HC emissions

**Fig. 13:**
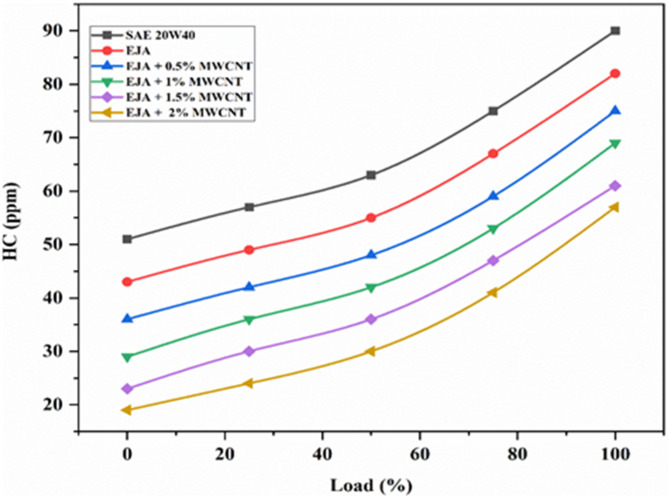
Variation of HC for SAE 20W40 and EJA various MWCNT nanoparticle concentrations.

The primary cause of hydrocarbon emissions is the inability to obtain the necessary amount of oxygen for full combustion, which may result from flame quenching near cylinder walls, fuel trapped in crevices, or poor air-fuel mixing. Inconsistent combustion or misfires also contribute to HC levels. As seen in Fig. 13, HC emissions increase as the load is increased and reaches a maximum at higher loads. It is also seen that HC emissions were found to be lower with the MWCNT additive than without it for all tested bio-lubricants. The reason is that MWCNT nanoparticles function as oxygen buffers for improved combustion. Mineral lubricant, if leaked into the combustion chamber, is not compatible with diesel fuel, and their combustion will lead to unburnt hydrocarbon emissions. On the other hand, vegetable-based lubricants are fully miscible with diesel fuel and they burn together releasing a high amount of heat energy. Due to this combustion will be well-organized and also may lead to lower UBHC emissions. The addition of nanoparticles will act as combustion promoters helping to lower ignition delay by promoting heat transfer within the combustion chamber. Hence, fuel will thoroughly mix with air, and combustion starts a little early, and complete burning will take place, which leads to lower UBHC emission. The percentage reduction in HC emission for full load for EJA + 2 wt% MWCNT is 30.48% and 36.66% as compared to EJA and SAE 20W40 respectively. Similarly, when the nanoparticle concentration is augmented from 0.5 to 2% HC emissions were reduced by 24%.

#### CO2 emissions

**Fig. 14:**
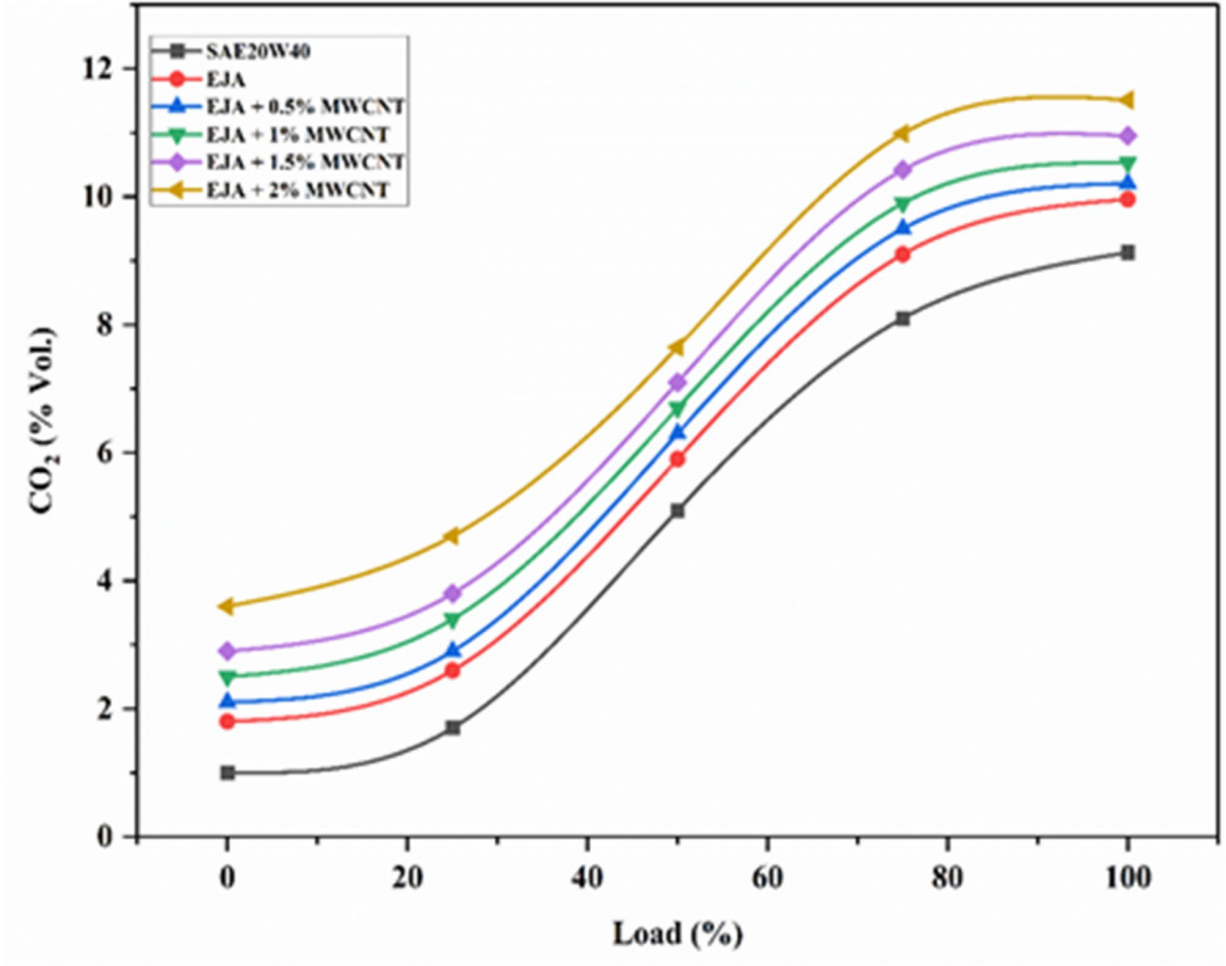
Variation of CO_2_ for SAE 20W40 and EJA with various MWCNT nanoparticle concentrations.

**Fig. 15:**
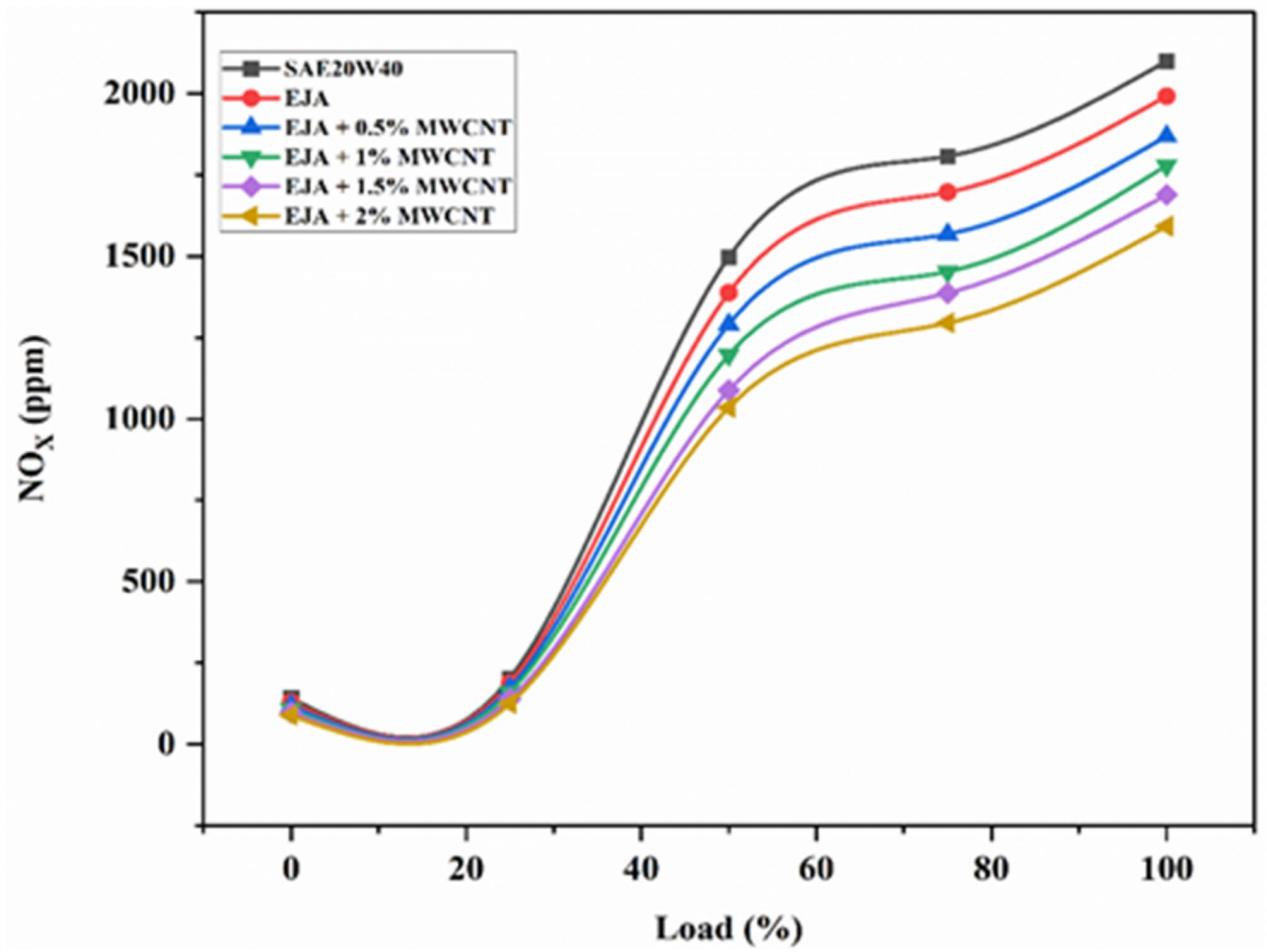
Variation of NO_X_ for SAE 20W40 and EJA with various MWCNT nanoparticle concentrations.

The changes in CO_2_ emissions in engine exhaust gas for SAE 20W40, EJA, EJA with MWCNT nanoparticles with the load are shown in Fig. 14. From Fig. 15 nanoparticle added bio-lubricants showed greater CO_2_ emissions, because extra oxygen present in the molecular structure of bio lubricant favour the combustion process and converting the CO present to CO_2_. Also, any nanoparticles accumulated at the combustion chamber will accelerate the chemical delay and hence reduces the ignition delay, which further helps for better conversion to CO_2_. The percentage increase in CO_2_ emission for full load for EJA + 2 wt% MWCNT is 15.56% and 26.06% as compared to EJA and SAE 20W40 respectively. In addition, there was an increase of CO_2_ by 12.73% when the MWCNT concentration is raised from 0.5 to 2%.

#### NOx emissions

Nitrogen oxide is the generalized term for NO and NO_2_ given with the formulae of NO_x_. High combustion temperatures (1800 K) cause nitrogen molecules to lose their strong triple bond, dissociate into their atomic states, and engage in several reactions with oxygen to produce thermal NO_x_. High temperatures inside engine combustion chambers cause NO_2_ from the surrounding air to break down, releasing various nitrogen oxides when it reacts with oxygen. This is the cause of NO_x_ emissions. A good lubricant with a higher thermal capacity can help regulate them by reducing the reaction that leads to the creation of NO_x_. Figure 15 displays the variation in NO_x_ emission for the tested lubricants. When SAE 20W40 lubricant is used, the engine temperature rises because of incomplete combustion, increasing NO_x_ emissions. Bio-lubricants in epoxidized form produce less NO_x_ than SAE 20W40 lubricant because of improved heat management. Due to the improved combustion and reduced after burning, the average cylinder temperatures might be reduced. When nanoparticles are added to the bio-lubricant, it can make combustion more efficient. This happens because the nanoparticles help transfer heat between the air and fuel faster, which reduces the time it takes for combustion to start. As a result, the cylinder temperature stays lower than it would with mineral oil lubricants. This reduces emissions of NO_x_, even though the bio-lubricants have higher oxygen content, the positive influence of the nanoparticle addition will negate the availability of extra oxygen which will have a positive effect on NO_x_ reduction. The percentage reduction in NO_x_ emission for full load for EJA + 2 wt% MWCNT is 20.08% and 24.15% as compared to EJA and SAE 20W40, respectively. When the nanoparticle concentration is increased from 0.5 to 2% NO_x_ emissions were reduced by 14.82%.

#### Smoke emissions

**Fig. 16:**
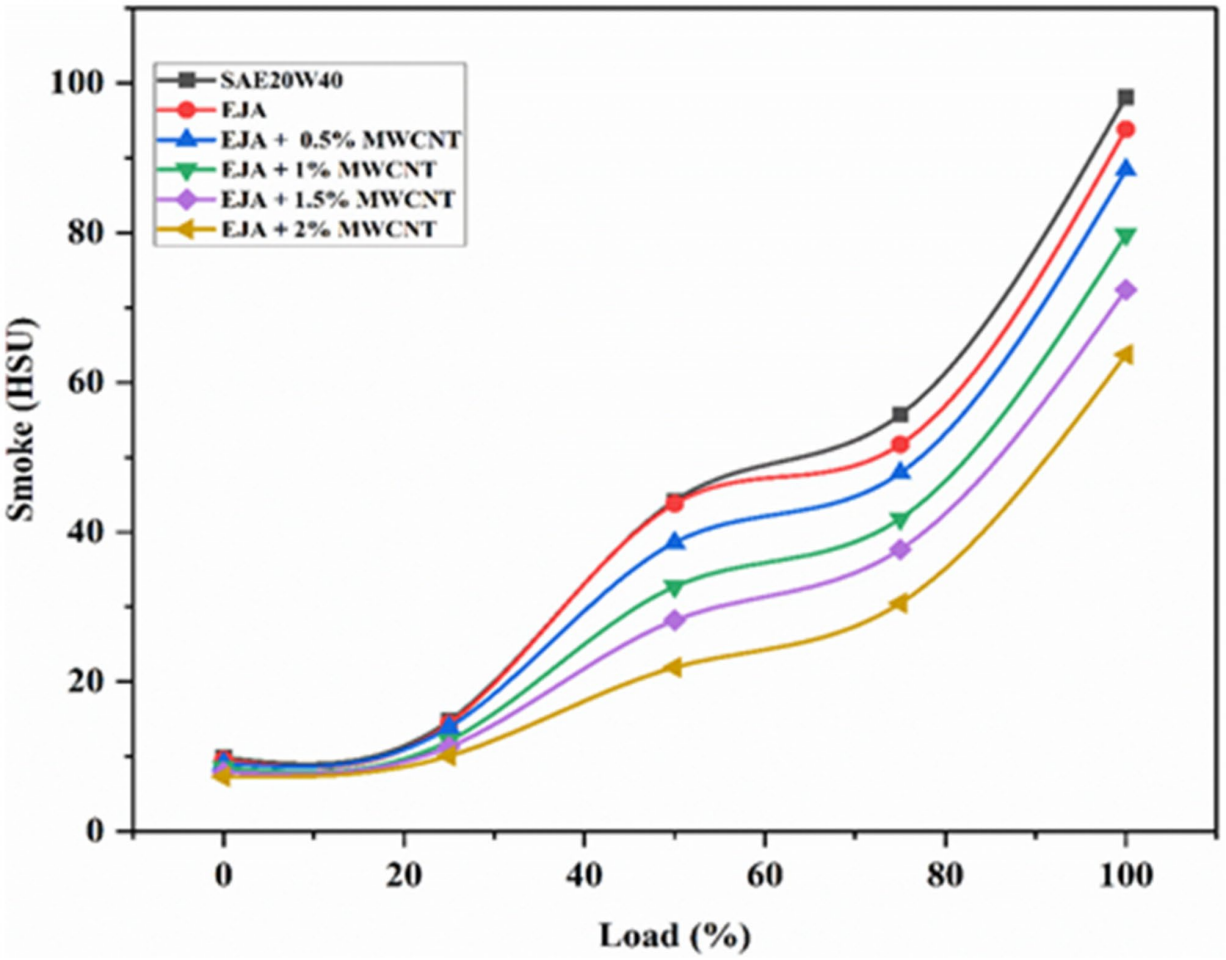
Variation of Smoke for SAE 20W40 and EJA with various MWCNT nanoparticle concentrations.

Particles of unburned carbon produced during the first stage of combustion make up smoke. The primary causes of soot production are the quenching activity that is taking place close to the cylinder wall and the lack of oxygen. Upon using the mineral lubricants, carbon particles are deposited at the wall’s surface because of quenching, which prevents heat from transferring. Due to this, fuel consumption rises because of increased frictional losses and decreased power production. When there is a significant air deficit, smoke is released. These effects will be reduced by using vegetable-based lubricants due to their properties such as superior detergency, dispensation, and higher oxygen availability. It can be seen in Fig. 16, for every lubricant that has been tested, it has been found that smoke opacity increases with load. It is higher for SAE 20W40 than for the bio-lubricant based on EJA. Further, one important catalytic factor in reducing the development of soot is the inclusion of MWCNT nanoparticles. The inclusion of MWCNT nanoparticles to the bio-lubricant causes a decrease in the temperature at which carbon activates. The oxidation of soot particles to CO_2_ can occur at this temperature leading to better combustion. EJA + 2 wt% MWCNT, hence lessens the production and emission of smoke. From Fig. 16, the nanoparticle added bio-lubricants i.e. EJA + 2 wt% MWCNT produces less smoke than the neat epoxidized bio-lubricants because of the increased lubricity. The percentage reduction in Smoke for full load for EJA + 2 wt% MWCNT is 32.08% and 35.06% as compared to EJA and SAE 20W40 respectively and 27.94% as compared to the emissions at 0.5% of nanoparticles.

Tables 5 and 6 show a summary of the engine performance and emissions for the following lubricant / bio-lubricant samples - SAE 20W40, EJA, and EJA-MWCNT. Performance and emission characteristics are compared with the literature and are shown in Table 7. It appears that the utilization of bio-lubricants in diesel engines has yielded promising results, as evidenced by the observed trend in performance parameters and emissions. Not only has there been a performance improvement, but there has also been a quantitatively significant reduction in emissions compared to what has been reported in published literature.

This suggests that the use of bio-lubricants holds the potential for enhancing both the efficiency and environmental friendliness of diesel engines. Further research and development in this area could lead to even greater advancements in engine technology and sustainability.

**Table 5:** Engine performance for SAE 20W40, EJA and EJA + 2 wt% MWCNT.

Lubricant/bio-lubricants	Friction power (kW)	BTE (%)	T_exhaust_ (K)
SAE 20W40	2.3	32.04	589
EJA		32.63	579
EJA + 2 wt% MWCNT	1.4	34.12	527

**Table 6:** Emissions for SAE 20W40, EJA and EJA + 2 wt% MWCNT.

Emissions	Lubricant/bio-lubricants		
	SAE 20W40	EJA	EJA + 2% MWCNT
CO (% Vol.)	1.61	1.49	0.99
HC (ppm)	90	82	57
CO_2_ (%Vol.)	9.13	9.96	11.51
NOx (ppm)	2099	1992	1592
Smoke (HSU)	98.1	93.8	63.7

**Table 7:** Comparison of performance and emission characteristics with the literature.

References	Bio-lubricants	Observations
Bekal and Bhat^14^	Pongamia oil	The increase in friction power was by 2.63% by using Pongamia oil as a lubricant The reduction in BSEC was 4.2% and the increase in BTE was found to be 4.29%. at medium load conditions At high loading conditions, an increase in BTE was found to be 1.29%
Kumar and Dinesha^15^	Gingelly oil	Esterified gingelly oil had the highest BTE and lowest BSFC when compared with neat gingelly and mineral oil. An increase in BTE was found to be 2.49% by using esterified gingelly oil as compared with mineral oil. At full load condition, reduction in NO_x_ and CO emissions were found to be 11.25 and 77.27% respectively.
Sarma et al.^22^	Racer-4 lubricant + Cu nanoparticles	Nano lubricants dispersed with nano Cu in 0.05, 0.1, and 0.2% mass fractions produced the improved BTE. When 0.05% Cu nano is added to the Racer-4 lubricant, the brake thermal efficiency increases by 4–5%. With the addition of 0.05% Cu to the lubricant, the reduction in HC, CO_2,_ and NO_x_ emissions was found to be 8.04%, 16.14 and 60.56% respectively.
Present study	Epoxidized jatropha + MWCNT	The reduction in friction power was by 39.13% as compared to SAE 20W40. The increase in BTE for EJA + 2 wt% MWCNT is 6.49% as compared to SAE 20W40. Engine experiments showed that the reduction in HC, CO, and smoke emission levels for EJA + 2 wt% MWCNT were 36.66%, 38.5%, and 35% respectively as compared to SAE 20W40.

## Conclusions

Based on the experimental research conducted by utilizing bio-lubricants (with and without nanoparticles) on diesel engine performance and characteristics, the following conclusions are drawn.

- The friction power obtained for EJA + 2 wt% MWCNT bio-lubricant was 1.25 kW. The percentage reduction in friction power for EJA + 2 wt% MWCNT bio-lubricant was found to be 39.13% as compared to SAE 20W40.
- The brake thermal efficiency was found to be highest for MWCNT nanoparticle additive bio-lubricant as compared to SAE 20W40. The percentage increase in BTE for EJA + 2 wt% MWCNT is 4.56% and 6.49% as compared to EJA and SAE 20W40 respectively.
- The percentage reduction in BSEC for EJA + 2 wt% MWCNT bio-lubricant was found to be 4.36% and 6.11% compared to EJA and SAE 20W40 respectively. T_exhaust_ was found to be lowest for EJA + 2 wt% MWCNT bio-lubricant with 10.52% than SAE 20W40.
- Experimental investigations revealed that EJA + 2 wt% MWCNT showed a reduction of CO emission by 38.50% as compared to SAE 20W40. Engine experiments showed that the reduction in HC emission levels for EJA + 2 wt% MWCNT was 36.66% as compared to SAE 20W40. The percentage increase in CO_2_ emission for EJA + 2% MWCNT was 26.06% as compared to SAE 20W40.
- For a combination of EJA + 2 wt% MWCNT bio-lubricant, the reduction in NO_x_ emission was found to be 24.15% as compared to SAE 20W40. The percentage reduction in Smoke emission for EJA + 2 wt% MWCNT bio-lubricant was 35.06% as compared to SAE 20W40.
- When the nanoparticle concentration is increased from 0.5 to 2% BTE increases by 3.61% whereas HC, CO, and smoke emissions are reduced by 24%, 27.20%, and 27.94% respectively.

The above studies revealed that the addition of nanoparticle additives to bio-lubricants in a diesel engine improves the engine efficiency by lowering the friction power and also helps in reducing the emissions compared to mineral lubricant. Overall, the use of bio-lubricants is a great way to promote sustainability and protect our planet.

## Data Availability

Data that support the findings of this study are available from the corresponding author, Dr Shiva Kumar, upon reasonable request.

## Abbreviations

EJA: Epoxidized jatropha
MWCNT: Multi walled carbon nanotubes
EJA + 0.5 wt% MWCNT: Epoxidized jatropha with 0.5% of multi walled carbon nanotubes
EJA + 1 wt% MWCNT: Epoxidized jatropha with 1% of multi walled carbon nanotubes
EJA + 1.5 wt% MWCNT: Epoxidized jatropha with 1.5% of multi walled carbon nanotubes
EJA + 2 wt% MWCNT: Epoxidized jatropha with 2% of multi-walled carbon nanotubes
H_2_SO_4_: Sulphuric acid
FP: Friction power
BTE: Brake thermal efficiency
BSEC: Brake specific energy consumption
BSFC: Brake specific fuel consumption
SFC: Specific fuel Consumption
TFC: Total fuel consumption
CV: Calorific value
CO: Carbon monoxide
CO_2_: Carbon dioxide
NO_x_: Oxides of nitrogen
HC: Hydrocarbon
